# The complete mitochondrial genome of the snakeskin gourami, *Trichopodus pectoralis* (Regan 1910) (Teleostei: Osphronemidae)

**DOI:** 10.1080/23802359.2017.1298418

**Published:** 2017-03-17

**Authors:** Han Ming Gan, Thumronk Amornsakun, Min Pau Tan

**Affiliations:** a School of Science, Monash University Malaysia, Selangor Darul Ehsan, Malaysia;; b Genomics Facility, Tropical Medicine and Biology Platform, Monash University Malaysia, Selangor Darul Ehsan, Malaysia;; c Fisheries Technology Program, Department of Technology and Industries, Faculty of Science and Technology, Prince of Songkla University, Pattani Campus, Pattani, Thailand;; d School of Fisheries and Aquaculture Sciences, Universiti Malaysia Terengganu, Terengganu, Malaysia;; e Institut Bioteknologi Marin (IMB), Universiti Malaysia Terengganu, Terengganu, Malaysia

**Keywords:** *Trichopodus pectoralis*, mitogenome sequencing, Malaysia, Vietnam, Thailand

## Abstract

We sequenced and assembled three whole mitogenome sequences of the commercially important snakeskin gourami *Trichopodus pectoralis* isolated from Malaysia (introduced), Viet Nam (native) and Thailand (native). The mitogenome length range from 16,397 to 16,420 bp. The final partitioned nucleotide alignment consists of 14,002 bp and supports the monophyly of the genus *Trichopodus* (95% ultrafast bootstrap support) with *T. trichopterus* forming a sister group with the members of *T. pectoralis.*

The snakeskin gourami *Trichopodus pectoralis* commercially used as ornamental and food fish, and it is the largest and most significant species among the trichogastrids. It is native to the central plain of Thailand, extending from Chao Phraya Basin to Cambodia and Vietnam, Laos (Boonsom 1985), but, it is now prevalent across Southeast Asia countries due to numerous past introductions. Three snakeskin gourami specimens were collected from Vietnam, Thailand, and Malaysia, and stored at the Institute of Oceanography, Universiti Malaysia Terengganu, with voucher ID UMTGen01310–01312. Genomic DNA (gDNA) extraction from ∼20 mg of fin clip was performed using the Solokov protocol (Sokolov 2000). Approximately 100–200 ng of purified gDNA was subsequently sheared to 500 bp using a M220 focused-ultrasonicator (Covaris, Woburn, MA), processed using NEBNext Ultra DNA library prep kit and sequenced on the MiSeq desktop sequencer (Illumina, San Diego, CA) located at the Monash University Malaysia Genomics Facility using a run configuration of 2 × 250 bp. For isolates UMTGen01311 and UMTGen01312, mitogenome assembly was performed using MITObim v 1.8 (Hahn et al. 2013), using the COX1 gene fragment of the species as the bait for iterative mapping assembly. For isolate UMTGen01311, the complete mitogenome of isolate UMTGen01312 was used instead of the bait for reference-based assembly. Each mitogenome was circularized (Gan et al. 2014) and annotated using MitoAnnotator (Iwasaki et al. 2013).

Protein coding genes (PCGs) were partitioned and aligned based on gene and codon position using TranslatorX (Abascal et al. 2010) while the 12S and 16S ribosomal RNA genes aligned using Muscle, generating 41 nucleotide alignments (13 PCGs x 3 codon positions +2 rRNA). Each alignment was subsequently trimmed with TrimAl version 1.9 (Capella-Gutiérrez et al. 2009) and concatenated. Best-fit partitioning scheme was calculated using ModelFinder version 1.5.3 (Nguyen et al. 2015) and a maximum-likelihood (ML) tree was constructed with 1000 ultrafast bootstrap approximation option. The consensus tree was visualized using FigTree 1.4.2 (http://tree.bio.ed.ac.uk/software/figtree/). Additional nucleotide similarity search to verify the taxonomy of *Trichopodus microlepis* (NC_027238) was performed using BlastN (Altschul et al. 1997).

ModelFinder identified five partitions as the best-fit partitioning scheme as follows (-# after gene name indicates codon position): Partition 1 = 12S and 16S rRNA; Partition2 = ATP6-1,NAD3-1,NAD4-1,COB-1,NAD4L-1,NAD1-1,NAD2-1,ATP8-1, NAD5-1,COX1-1,COX2-1,COX3-1,NAD6-1; Partition3 = ATP6-2, NAD4-2,NAD6-2,NAD2-2,ATP8-2,NAD5-2,COB-2,NAD4L-2,NAD1-2, NAD3-2,COX2-2,COX1-2,COX3-2; Partition 4 = ATP6-3,NAD1-3,NAD3-3,NAD5-3,NAD4-3,COX3-3,NAD4L-3,COB-3,ATP8-3,COX2-3, NAD2-3,COX1-3 and Partition 5 = NAD6-3.

The final partitioned nucleotide alignment consists of 14,002 bp length and supports the monophyly of the genus *Trichopodus* (95% ultrafast bootstrap support) with *Trichopodus trichopterus* forming a sister group with members of *Trichopodus pectoralis* reported in this study (Figure 1). It is also worth noting that GenBank entry NC_027238 was initially submitted as *Trichopodus microlepis*, however the COX1, COB, and 12S rRNA genes of this mitogenome was consistently lower than 90% identity to similar gene fragments from vouchered specimens in NCBI database (Accession codes: KU569058, KU569059, KF805359, KF805360, AY763711 and AY763757), suggesting potential misidentification and the need to sequence the whole mitogenome of a correctly identified *Trichopodus microlepis* specimen.

**Figure 1.**
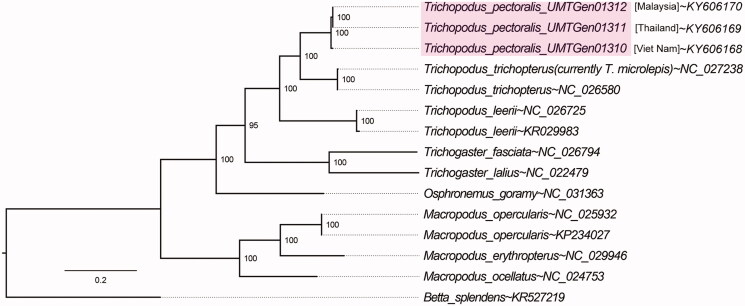
Evolutionary relationships of *Trichopodus pectoralis* reported in this study (shaded) as inferred from maximum-likelihood estimation based on 13 mitochondrial protein-coding genes and 2 ribosomal RNA genes with best-fit partitioning scheme with *Betta splendens* rooted as the outgroup. NCBI accession numbers are provided next to tilde (∼) symbols. Numbers at nodes indicate Ultrafast Bootstrap support and branch lengths indicate the number of substitutions per site.
